# Drug-related problems among breastfeeding patients treated for depressive spectrum disorders

**DOI:** 10.3389/fphar.2024.1440681

**Published:** 2024-07-23

**Authors:** Karolina Morze, Edyta Szałek, Magdalena Waszyk-Nowaczyk

**Affiliations:** ^1^ Department of Clinical Pharmacy and Biopharmacy, Poznan University of Medical Sciences, Poznan, Poland; ^2^ Pharmacy Practice and Pharmaceutical Care Division, Department of Pharmaceutical Technology, Poznan University of Medical Sciences, Poznan, Poland

**Keywords:** drug-related problems, lactation, depressive spectrum disorders, breastfeeding, depression

## Abstract

**Introduction:**

Depressive spectrum disorders are common and can hinder breastfeeding success. While medications typically pose minimal risk, the concerns persist. This is the first study that investigates the prevalence and characteristics of drug-related problems among breastfeeding mothers with depressive spectrum disorders. We analyzed those problems to understand their nature, severity, and contributing factors. Additionally, we evaluated the outcomes of pharmacist-led interventions in reducing them. Understanding drug-related problems is crucial for informing evidence-based practices to optimize both maternal mental health and breastfeeding success.

**Materials and methods:**

This prospective observational study was conducted at a specialized pharmacy office in Poznan, Poland, which focuses on lactation support and medication consultations. 47 breastfeeding patients were enrolled. Pharmaceutical consultations were conducted according to Joint Commission of Pharmacy Practitioners Pharmacists’ Patient Care Process standards. Novel MILC Questionnaire was used for efficient and optimal pharmaceutical interview. Drug-related problems were assessed basing on PCNE Classification System version 9.1. For adverse events in lactation, MedDRA v27 nomenclature was used; for causality, Naranjo Scale and LCAT were utilized. CTCAE was used for grading.

**Results:**

Among the 47 patients, pharmacist identified 49 medication-related problems, with inadequate treatment effect due to underdosing or not taking the medication at all being the most common (57.1%). Pharmacist interventions focused on medication safety information and counseling. Overall, 78.7% of patients accepted these interventions, resulting in problem resolution for 71.4%. Twelve mothers (25.5%) reported adverse events in their infants, but after causality evaluation, only four (8.5%) might have been linked to maternal medication. None required medical intervention beyond one hospitalization for a serious adverse event possibly connected to maternal medication.

**Conclusion:**

The study identified high rates of drug-related problems among breastfeeding mothers with depression, primarily due to non-adherence. Pharmacist interventions significantly improved DRP outcomes. Adverse events were reported, but most were mild and did not require intervention. Our findings suggest that lactating mothers with depressive spectrum disorders may benefit from pharmacist-led support to optimize treatment adherence and address medication safety concern.

## 1 Introduction

Extensive evidence demonstrates the long-term health benefits of exclusive breastfeeding for both the offspring and the mother (Brown et al., 2014; Horta, de Mola, Victora, 2015; World Health Organization, 2017; Perez-Escamilla et al., 2023). Numerous studies have established its association with reduced risk of various illnesses, including obesity, type 2 diabetes, ovarian cancers, and cardiovascular diseases (Louis-Jacque and Stuebe, 2020; Perez-Escamilla et al., 2023).

Despite established protective benefits associated with prolonged breastfeeding, achieving the minimum recommended duration of 6 months (Brown et al., 2014; World Health Organization, 2017; Inano et al., 2021) remains a challenge for numerous parents globally. This incomplete realization of lactation goals may not confer the same health advantages as extended breastfeeding.

Approximately 10% of mothers who discontinue breastfeeding before the recommended 6 months cite medical reasons as a motivator for the cessation (Brown et al., 2014; Inano et al., 2021). Among medical issues, depressive spectrum disorders represent the most common obstetrics complication (Rivi et al., 2020).

Postpartum women suffering from depression and anxiety disorders exhibit an increased risk of encountering breastfeeding difficulties. These difficulties may not only compromise breastfeeding duration but also potentially accelerate the onset of or exacerbate pre-existing depressive symptoms (Hoff et al., 2019; Jones et al., 2020; Rivi et al., 2020; Kim et al., 2022).

Prompt and accurate diagnosis and effective treatment of maternal mental health conditions are crucial for both optimizing maternal wellbeing and maximizing the chance for breastfeeding success, increasing the likelihood of achieving lactation goals for those mothers who intend to breastfeed.

While most commonly prescribed medications for depressive spectrum disorders pose minimal risk to breastfed infants with infrequent reports of adverse effects (Ito et al., 1993; Soussan et al., 2014; Sriraman et al., 2015; Anderson et al., 2016; Ahmandzai et al., 2022; Hale and Krutsch, 2023), mothers might still have concerns. Some healthcare professionals continue advocating for blanket breastfeeding cessation (Saha et al., 2015), prioritizing this approach over individualized risk-benefit assessments for each patient.

Facing disease symptoms and recommendations to discontinue breastfeeding due to medication use for mental illness presents mothers with a challenging dichotomy: prioritizing treatment adherence or continued breastfeeding. This potentially results in suboptimal outcomes in either domain, leading to a risk of premature termination of breastfeeding or drug-related problems (DRPs).

A DRP is defined as an event or circumstance involving drug therapy that actually or potentially interferes with desired health outcomes (Pharmaceutical Care Network Europe Association, 2020). DRPs constitute a significant barrier to achieving optimal therapeutic outcomes for patients. Recent literature indicates a potential association between DRPs and increased morbidity, mortality, and overall healthcare costs (Garin et al., 2021; Sheleme et al., 2021).

While research on depressive disorders treatment and lactation is extensive, studies specifically investigating DRPs in this population remain scarce. Studies exploring medication adherence, risk factors for DRPs, and their impact on both maternal and infant wellbeing are crucial for informing evidence-based clinical practice.

Primary Objective: This study aims to investigate the prevalence and characteristics of DRPs among lactating women receiving treatment for depressive spectrum disorders. In-depth analysis of the identified DRPs will be conducted to understand their nature, severity, and potential contributing factors.

Secondary Objective: This study will evaluate the outcomes of a pharmacist-delivered interventions in reducing DRPs in this patient population.

## 2 Materials and methods

This is a prospective observational study conducted among lactating women receiving medications for depressive spectrum disorders who registered for a pharmacist’s consultation between 1 September 2022 and 1 December 2023.

### 2.1 Study setting

This prospective observational study was conducted at a specialized pharmacy office in Poznan, which was the first such office in Poland, focusing on lactation support and medication consultations (Laktaceuta). This private service and consultation center provides professional guidance to patients seeking advice on medication use during breastfeeding. Services are delivered online through a dedicated web platform and include consultations, medication safety information, over-the-counter drug recommendations, drug safety monitoring, and identification and resolution of DRPs. The office is located in Poznan, but provides services to patients from all over Poland.

### 2.2 Platform access and services

Accessing the online platform requires registration on dedicated website. While registration is free, consultations and other services incur a fee. The platform is accessible on any internet-connected device.

### 2.3 Participants

From 1 September 2022 to 1 December 2023, a total of 457 patients from different locations in Poland registered for consultations. 47 were taking medications for depressive spectrum disorders that were diagnosed prior to the consultation, and this group of patients was included in this study.

### 2.4 Study design

**FIGURE 1:**
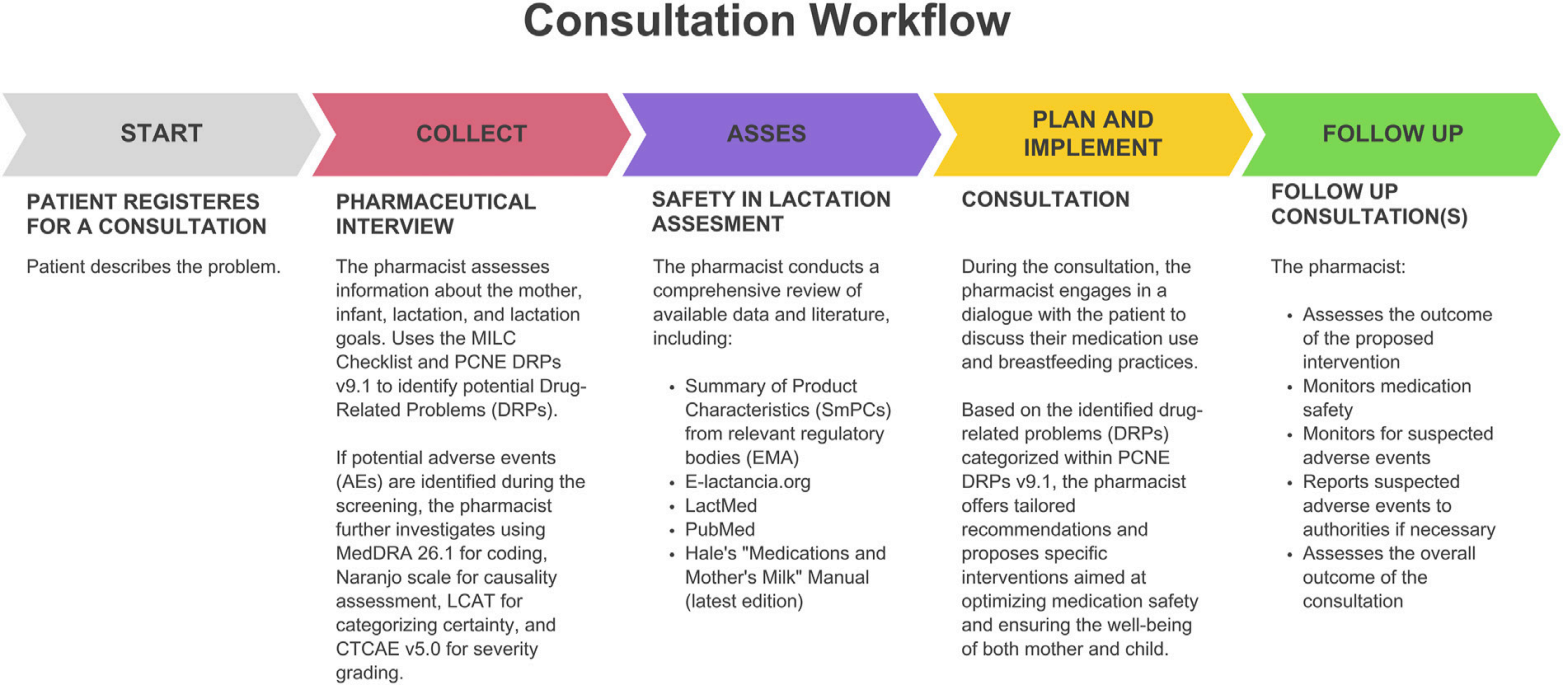
Pharmaceutical consultation workflow.

At a Specialized Pharmacy Office “Laktaceuta” consultations are conducted according to Joint Commission of Pharmacy Practitioners Pharmacists’ Patient Care Process standards (Joint Commision of Pharmacy Practicioners, 2014) that consist of a five-step process of Collecting information, Assessing, Planning, Implementing, and Follow up (Figure 1). Interdisciplinary cooperation with specialists from other fields was implemented when necessary.

After registration, patients went through a complete Consultation Workflow (Figure 1).

**FIGURE 2:**
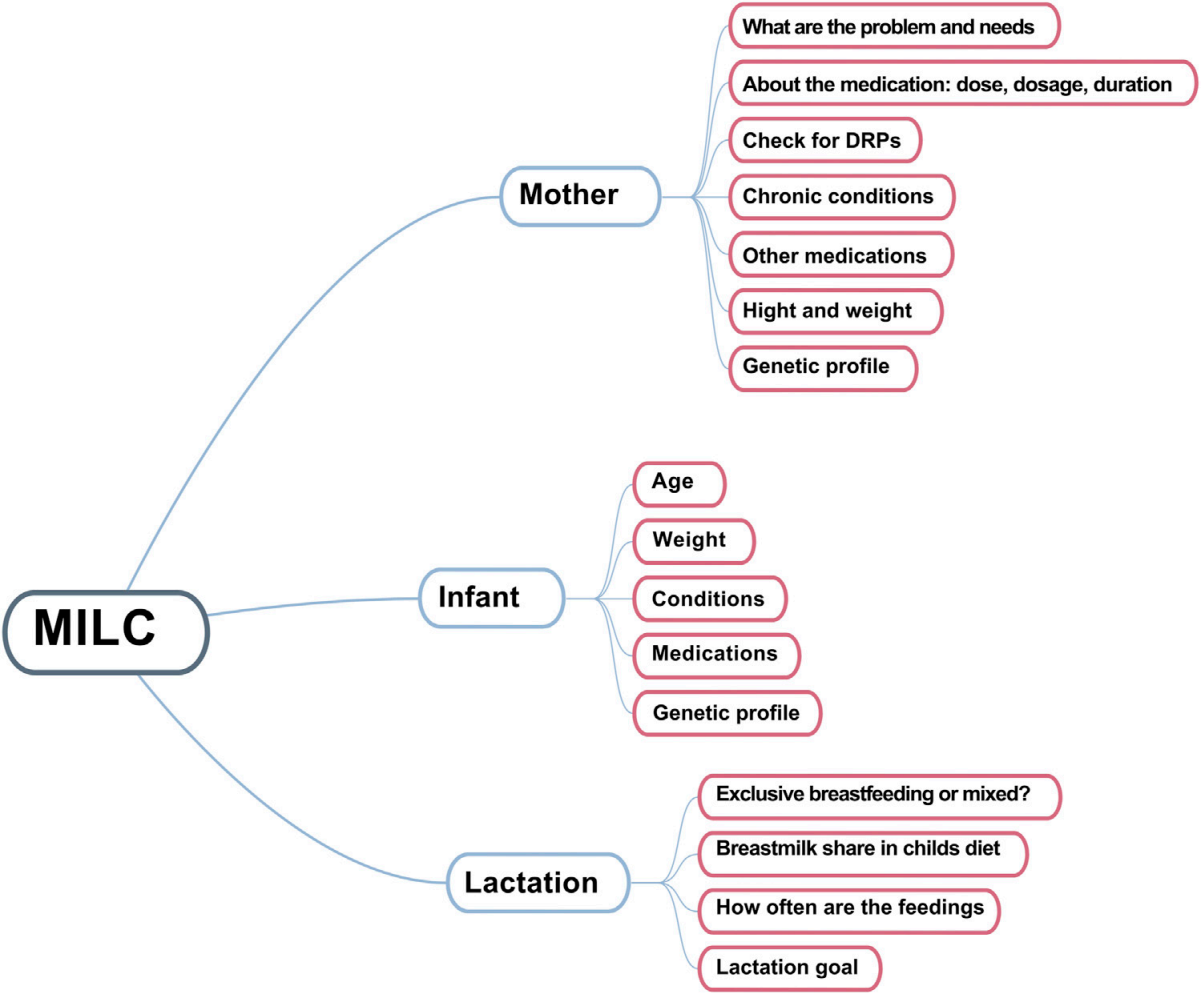
MILC questionnaire.

Data collection during the pharmacist interview relied on a novel, author-designed tool, that incorporates factors specific to breastfeeding—MILC questionnaire (Figure 2). DRPs were identified utilizing the PCNE Classification System version 9.1. (Pharmaceutical Care Network Europe Association, 2020).

### 2.5 The MILC questionnaire

The MILC questionnaire is a novel instrument designed to efficiently assess drug safety in the context of breastfeeding (Figure 2). It comprises three sections:

Mother (M): This section collects information pertaining to the mother, including presenting complaints, medical history, diagnoses, comorbidities, current medication use, and genetic profile if available. Additionally, pharmacist includes the identification of potential DRPs in this section.

Infant (I): This section focuses on the breastfed child, gathering data on age, weight, medical history, current medications, and genetic profile if available.

Lactation (LC): This section explores the mother’s breastfeeding goals, history, and current practices.

The MILC questionnaire is in a form of a checklist, and facilitates the collection of essential information for the assessment of medication safety in breastfeeding. Such information includes, but is not limited to, maternal dose and dosage, treatment duration, estimated infant milk intake, child’s developmental stage, and health status. This comprehensive approach ensures efficient patient needs assessment and identification of potential or existing DRPs without overburdening the mother.

### 2.6 PCNE classification 9.1

This system utilizes a five-domain structure. The Domain Problems (P) categorizes identified DRPs into three subdomains: P1 Treatment Effectiveness (addressing issues with the medication’s intended effect), P2 Treatment Safety (concerned with potential or experienced adverse events), and P3 Other (encompassing DRPs not falling under the previous categories).

Further details are provided by the Domain Causes (C), consisting of nine subdomains that explore potential DRP originators. Domain Planned Interventions (I) outlines five possible solutions a pharmacist can propose. Domain Intervention Acceptance (A) captures patient acceptance or refusal of these interventions, reflecting real-world implementation. Finally, Domain Status of the DRP (O) tracks the identified DRPs’ outcomes and intervention effectiveness through four subdomains: the intervention’s success in resolving the DRP and any additional details regarding the outcome.

A comprehensive evaluation was undertaken to identify and categorize drug-related problems (DRPs) among the patients. Data collection included information from initial registration inquiries, standardized interviews, and in-depth interviews. Co-administration of other medications, supplements, the presence of concurrent illnesses, lifestyle factors, and other relevant details were considered during DRP identification and categorization.To minimize potential bias in DRP categorization, specific cases from this study were consulted with the PCNE working group, ensuring accuracy and consistency through their expertise.

### 2.7 Drug safety in lactation

During the process of consultation, the following clinical decision-making tools and drug databases were used: LactMed (Drugs and Lactation Database, 2006) and E-Lactancia (E-Lactancia.org). Literature and data review was based also on Safety Score (Uguz, 2021), PubMed search, drug Summary of Product Characteristics—SmPCs, and “Medications and Mothers Milk” Manual 2023 (Hale and Krutsch, 2023).

### 2.8 Adverse events

Adverse event (AE) identification relied primarily on maternal descriptions, potentially introducing subjectivity. To mitigate this potential bias, a comprehensive evaluation was conducted upon identifying a suspected AE. This evaluation encompassed factors pertaining to the mother, lactation process, and the infant, and aimed to establish a clear link between the medication and the reported effect, ensuring accurate attribution of causality.

Causality evaluation included a detailed interview to assess potential contributing factors in both the mother and infant. Factors investigated encompassed co-morbidities, chronic illnesses, concomitant medications (including supplements and herbal preparations), recent life changes, enviornmental factors, and any other elements that might influence the observed AE. Prior to study initiation, these factors were comprehensively discussed with a neonatologist, a pediatrician, and a midwife to ensure a holistic approach to identifying potential contributors to the AEs. Whenever possible, medical records were retrieved, including past medical history, physical examination findings, and physician visit summaries. To ensure data clarity and consistency, all AEs were classified using standardized MedDRA codes (version 27).

MedDRA^®^ the Medical Dictionary for Regulatory Activities terminology is the international medical terminology developed under the auspices of the International Council for Harmonization of Technical Requirements for Pharmaceuticals for Human Use (ICH).

Specific cases from this study were consulted with the MedDRA coders and training staff, ensuring accuracy and consistency through their expertise. Severity grading of adverse events (AEs) adhered to the Common Terminology Criteria for Adverse Events (CTCAE) scale version 5.0. Causality assessment of DRPs employed two established tools: the Naranjo scale (Naranjo et al., 1981) and the Liverpool Causality Assessment Tool (LCAT) (Gallagher et al., 2011).

## 3 Results

### 3.1 Participants

The participants of this study were 47 breastfeeding women nursing their children. These parents had prior diagnoses within the depressive spectrum, encompassing a range of conditions. Specifically, diagnoses included anxiety disorders, neurotic disorders, mixed anxiety-neurotic disorders, sleep disorders (including insomnia), and depressive disorders (comprising general depression, depressive episodes, and postpartum depression). Prior to pharmacist consultation, each patient had received medical evaluation and treatment recommendations from a physician.

A summary of the studied population data from the MILC questionnaire is presented in Table 1. 10 mothers had chronic conditions that included migraines, thyroid inflammation, recurrent HSV infection, tetany, Hashimoto, anemia, sclerosis multiplex, Crohn’s disease, spongy kidney. 14 of all of the mothers were taking medications daily either for chronic conditions or dedicated supplements for lactating parents.

**TABLE 1:** Maternal and infant data based on the MILC Questionnaire including lactation (S, arithmetic mean; SD, Standard Deviation; DRP, Drug-Related Problem; BMI, Body Mass Index; CYP, Cytochrome P450).

					Number of cases	%
Mothers				47		
Mother’s data	S	SD	Patient’s needs	Drug information and safety assessment	26	55.3
Weight	63.37 kg	15.27		Second opinion	20	42.6
Height	168,7 cm	6.49		Adverse event suspected	1	2.1
BMI	23.03	3.25	**Check for DRPs**		47	
				None	5	10.6
				P1 Treatment Effectiveness	32	68.1
				P2 Treatment safety	10	21.3
				P3 Other	0	0
			**Drugs concerned**	Monotherapy		
				Citalopram	2	4.3
				Duloxetine	2	4.3
				Escitalopram	5	10.6
				Fluoxetine	2	4.2
				Opipramole	1	2.1
				Paroxetine	1	2.1
				Sertraline	24	51
				Polytherapy		
				Escitalopram, fluvoxamine, lorazepam	1	2.1
				Escitalopram, mianserin	1	2.1
				Escitalopram, oxazepam	1	2.1
				Hydroxy zine, doxylamine	1	2.1
				Hydroxyzine, capropril	1	2.1
				Mirtazepine, mianserin	1	2.1
				Sertraline, escitalopram	1	2.1
				Sertraline and not specified	1	2.1
				Sertraline, hydroxy zine	1	2.1
				Sertraline, mianserin, quetiapine	1	2.1
			**Chronic conditions**	Yes	10	
				Crohn’s disease	1	2.1
				*Helicobacter pylori* infection	1	2.1
				Multiple sclerosis	1	2.1
				Migraine	1	2.1
				Recurrent HSV	1	2.1
				Spongy kidney	1	2.1
				Tetany	1	2.1
				Thyroid diseases	3	6.4
				No	37	78.7
			**Medications**	Yes	14	29.8
				Acyclovir	1	2.1
				Capt opril	1	2.1
				Contraceptives oral	2	4.2
				Ergotamine	1	2.1
				Levothyroxine and supplements	2	4.2
				Supplements only	7	14.9
				No	33	70.2
			**Genetic profile**	Genetic profile for CYP polymorphism tested	1	2.1
**Infants**				47		
Infant’s data	S	SD	**Conditions**	Yes	5	10.6
Age	7.97 months	8.85		Atopic dermatitis	1	2.1
Weight	7710g	2906		Low birth weight	1	2.1
				Increased muscle tension	1	2.1
				Neonatal jaundice	1	2.1
				Trisomy 21, atrioventricular septal defect	1	2.1
				No	42	89.4
			**Medications**	Yes	6	12.8
				Lisinopril	1	2.1
				Supplements	5	10.6
				No	36	76.6
				No data	5	10.6
**Lactation**					47	
			**Breast milk share in the child’s diet**	About 100%	27	57.4
				About 75%	6	12.8
				About 50%	6	12.8
				Up to 25%	6	12.8
				No data	2	4.2

The study cohort included no twins or instances of concurrent breastfeeding of siblings with different ages (tandems). The average infant age was 9 months, with a range extending from 1 day to 41 months (>3 years). Five children presented with pre-existing medical conditions: atrioventricular septal defect and Down syndrome, neonatal jaundice, atopic dermatitis, and increased muscle tone. Daily supplementation or medication use was reported for six infants.

In one mother-child pair, genotyping revealed CYP2D6 and CYP2C19 wild-type status in the mother. Conversely, the child harbored a heterozygous 1*/4* allele variant for CYP2D6, classified as a “moderate” metabolizer.

The majority of participants breastfed exclusively, with human milk comprising 100% of the infant’s dietary intake. Introduction of solid foods occurred concomitantly with continued breastfeeding in older infants. Two mothers exclusively bottle-fed their infants with expressed breast milk.

A comprehensive pharmaceutical interview was conducted with 47 patients to assess their needs and concerns regarding medication use during lactation. The primary need identified, reported by 26 patients (55.3%), was medication safety evaluation. Twenty patients (42.6%) sought a second opinion on medication safety in the context of breastfeeding, while one patient (2.1%) desired consultation regarding a potential adverse drug event.

### 3.2 Drug-related problems

**FIGURE 3:**
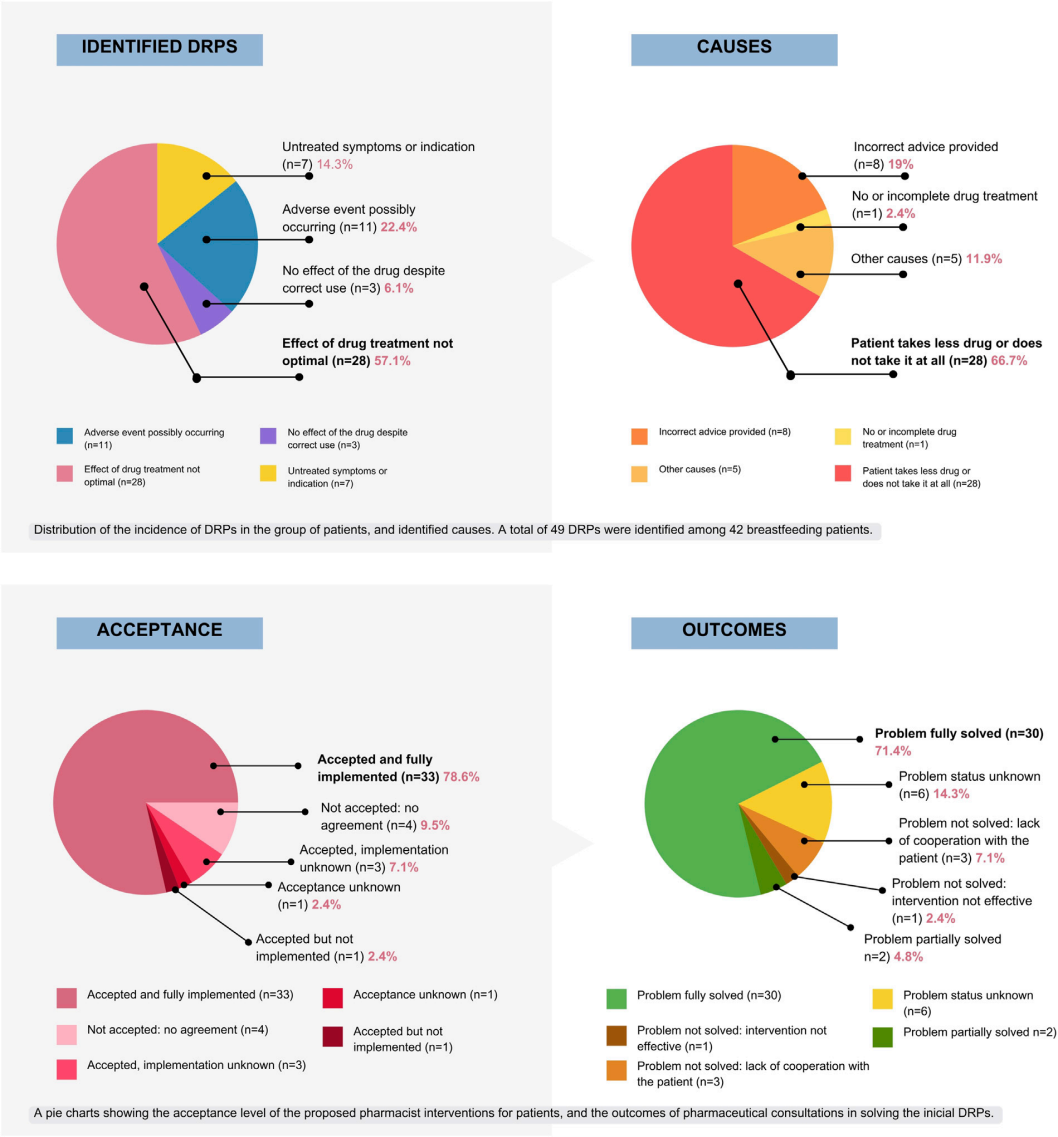
Graphical presentation of DRPs distribution among the studied population, identified causes, level of acceptance and consultation outcomes that followed pharmacist’s proposed interventions, based on PCNE DRP v 9.1 (Pharmaceutical Care Network Europe Association, 2020).

During initial interview with 47 registered patients, the pharmacist identified a total of 49 DRPs among 42 mothers. Five patients (10.6%) only required confirmation of drug information and had no problems regarding the treatment. 36 mothers (76.6%) had a single DRP identified and the remaining six patients (12.8%) presented with multiple DRPs. Table 2 and Figure 3 present gathered and analyzed data concerning identified DRPs, proposed interventions, intervention acceptance, and corresponding DRP outcomes for the study population based on PCNE Classification for drug-related problems V9.1 (Pharmaceutical Care Network Europe Association, 2020).

**TABLE 2:** Distribution of the occurrence of drug-related problems (DRPs) in the population of studied patients, identified causes of these problems, interventions proposed by the pharmacist, level of acceptance, implementation of interventions, and outcome of the consultation. Based on Pharmaceutical Care Network Europe Association (2020).

*Drug-Related Problem identified at the pharmaceutical interview (number of DRPs identified in each domain)*			*Causes (Codes v9.1, number of causes for this DRP)*			*Interventions proposed (Codes v9.1, number of interventions for this DRP, more than one for one patient)*			*Acceptance (Codes v9.1, number of cases with this acceptance)*			*Outcomes (Codes v9.1, number of cases with this outcome)*		
*One DRP occurring in the total of 36 patients*														
Code	Description	n	Code	Description	n	Code	Description	n	Code	Description	n	Code	Description	n
P1.2	Effect of drug treatment not optimal	23	C7.1	Patient intentionally takes less drug than prescribed or does not take the drug at all	21	I2.1	Patient (drug) counselling	23	A1.1	Intervention accepted and fully implemented	16	O1.1	Problem fully solved	16
			C5.2	Incorrect advice provided	1	I3.6	Drug started	20	A2.2	Intervention not accepted: no agreement	4	O0.1	Problem status unknown	3
			C1.5	No or incomplete drug treatment in spite of existing indication	1	I2.3	Patient referred to prescriber	2	A1.4	Intervention accepted, implementation unknown	1	O3.1	Problem not solved, lack of cooperation with the patient	3
						I3.2	Dosage changed	1	A1.3	Intervention accepted but not implemented	1	O3.3	Problem not solved, intervention not effective	1
									A3.1	Intervention proposed: acceptance unknown	1			
P1.3	Untreated symptoms or indication	7	C7.1	Patient intentionally takes less drug than prescribed or does not take the drug at all	4	I2.1	Patient (drug) counselling	7	A1.1	Intervention accepted and fully implemented	6	O1.1	Problem fully solved	6
			C5.2	Incorrect advice provided	3	I3.6	Drug started	5	A1.4	Intervention accepted, implementation unknown	1	O0.1	Problem status unknown	1
						I2.3	The patient was referred to the prescriber	2						

P2.1	Adverse drug event (possibly) occurring	6	C5.2	Incorrect advice provided	2	I2.1	I2.1 - Patient (drug) counselling	6	A1.1	Intervention accepted and fully implemented	5	O1.1	Problem fully solved	4
			C9.2	Other cause - not related to medication	4	I4.1	Other intervention (The pharmacist proposed taking the medication and breastfeeding without a pause)	2	A1.4	Intervention accepted, implementation unknown	1	O0.1	Problem status unknown	2
						I1.4	Proposed intervention consulted with a specialist	1						
***Multiple DRPs occurring in the total of 6 patients***														
P1.2, P2.1	Effect of drug treatment not optimal	3	C7.1	Patient intentionally takes less drug than prescribed or does not take the drug at all	3	I2.1	Patient (drug) counselling	6	A1.1	Intervention accepted and fully implemented	6	O1.1	Problem fully solved	4
	Adverse drug event (possibly) occurring		C5.2	Incorrect advice provided	2	I3.6	Drug started	6				O2.1	Initial problem partially solved	2
P1.1, P1.2, P2.1	No effect of drug treatment despite correct use	1	C9.2	Other cause - frequent drug changes	1	I3.2	Dosage changed	1						
	Effect of drug treatment not optimal					I3.1	Proposed drug changed to other (mirtazapine to mianserin)	1						
	Adverse drug event (possibly) occurring					I4.1	Other intervention (The pharmacist proposed taking the medication and breastfeeding with a pause)	1						
P1.1, P2.1	No effect of drug treatment despite correct use	1												
	Adverse drug event (possibly) occurring													
P1.1, P1.2	No effect of drug treatment despite correct use	1												
	Effect of drug treatment not optimal													

The most frequent DRP identified among the study population (57.1%) was inadequate therapeutic effect (P1.2, n = 28). The primary contributing factor to this DRP was non-adherence, defined as intentional underdosing or not taking the prescribed medication at all.

Seven identified DRPs were “untreated symptoms or indications” (14.3%), meaning the drug was not available or not prescribed despite indication.

While only one patient initially sought consultation for an adverse event (AE), pharmacist identified a total of eleven potential AEs during interviews, requiring further monitoring and follow up. Two mothers were unaware of AEs associated with selective serotonin reuptake inhibitor (SSRI) therapy initiation. In four cases, the identified symptoms were unlikely to be associated with the prescribed medications.

The causes were analyzed individually for each patient. In 66.7% of all cases, the patients intentionally underdosed or did not take the medication at all. In eight cases (19%), incorrect advice was provided regarding drug safety in lactation prior to the consultation. In five cases, there were other patient-related factors involved, like medication switching without proper supervision or early discontinuation due to no effect observed, that occurred in three mothers with multiple identified DRPs.

### 3.3 Safety in lactation assesment

Among 47 patients treated for depressive spectrum disorders, sertraline emerged as the most frequently prescribed medication (n = 28, 59.6%) either as a monotherapy (85.7%) or combined therapy involving concomitant medications (14.3%). Escitalopram followed in prevalence (n = 9), then citalopram (n = 2), duloxetine (n = 1), opipramol (n = 1), and paroxetine (n = 1). Regarding polytherapy, 37 patients (78.7%) received monotherapy, while 10 (21.3%) received two or more medications.

Comprehensive medication safety information for lactation was retrieved and verified through multiple sources. The essential information extracted from these sources for the consulted medications is summarized in suplemmentary material. A majority of the reviewed medications were classified as low-risk for breastfeeding according to established resources, including LactMed (Drugs and Lactation Database, 2006), E-lactancia.org, and Dr. Hale’s “Medications and Mother’s Milk”.

### 3.4 Interventions and acceptance

The primary interventions for DRPs consisted of pharmacist-led patient counselling (n = 42). It included drug safety information based on individual risk factors and up-to-date scientific data. A pharmacist also provided information and support to address patient concerns and anxieties regarding medication use during lactation. Pharmacist educated patients on proper medication use, potential side effects, and pediatric concerns.

Medication initiation was recommended in 31 cases (66%). Pharmacist referred four patients to their prescriber for further evaluation and decision-making. Pharmacist discussed one case directly with the prescriber to reach a joint decision, advised two patients to adhere to the prescribed full dose. Pharmacist proposed to switch medication in one case, recommended changes in breastfeeding practices to two patients to improve compliance (in one case) and minimize infant exposure (in the other case).

The interventions are structured in Table 2.

Among 42 patients with identified DRPs, pharmacist-proposed interventions were accepted by 37 (88.1%). Of those, 33 were fully implemented (89.2%), in three cases implementation was unknown (7.1%), in one case the intervention was not implemented (2.4%).

In four cases, no agreement was reached regarding the proposed interventions (9.5%). For one patient (2.4%), the intervention was recommended but ultimately the acceptance status remained unknown due to the lack of patient contact.

### 3.5 Outcomes

Among 42 patients with identified DRPs, pharmacist interventions resulted in complete problem resolution in 30 cases (71.4%). For two patients (4.8%), the problem was partially resolved. The problem was not solved due to lack of cooperation with the patient in three instances (7.1%), and in one case, the intervention was not effective (2.4%). Problem status was unknown in six cases—contact with the patients was lost (14.3%).

While we were unable to determine the final consultation outcomes (resolution of DRPs) for these six patients, we were able to collect complete data on their interviews and consultations and other relevant information. Therefore, this missing data has minimal impact on the overall results of the primary goal of this study.

### 3.6 Adverse events

During the pharmaceutical interview or follow-up, 23 out of 47 patients (48.9%) reported a total of 36 adverse events (AEs). Of these patients, some reported AEs related to themselves, lactation, or their babies (maternal AEs: n = 22; infant AEs: n = 12). Among the maternal AEs, 15 were classified as “probable,” which aligned with the expected side effects and information in the Summary of Product Characteristics (SmPCs) for those medications. Conversely, only one AE was classified as “probable” among the 12 that were identified in the infants.

In one case, the reported adverse event was not associated with the medication used for depressive spectrum disorder. Instead, it appeared to be related to the concurrent use of medication for lochia retention.

Most AEs were observed for sertraline (maternal AEs: n = 13, infant’s AEs: n = 10). The observed higher frequency of AEs associated with sertraline compared to other medications may be attributed to a selection bias within the study group. This bias arises from the fact that a larger proportion of participants received sertraline compared to other medications. Sertraline is considered the first-line medication for breastfeeding women, leading to its more frequent prescription.Table 3, 4 presents the occurrence of these events in mothers and their children (accordingly), described by the symptoms and coded using medDRA v 27. Causality was assessed using Naranjo scale (Naranjo et al., 1981), LCAT tool (Gallagher et al., 2011). Grading is also presented. Naranjo score and LCAT score were consistent only in nine out of 23 cases.

**TABLE 3:** This table details 17 cases where AEs were reported among mothers. Patients 13–17 reported AEs concerning lactation. Each case includes the name of the drug taken, dose taken at the moment the AE ocurred, a description of the adverse event using MedDRA terms (version 27). Each case was assessed for causality using both the Naranjo algorithm (Naranjo et al., 1981) the LCAT tool (Gallagher et al., 2011), and graded using the CTCAE scale. *In this case, the adverse event was probably linked to ergotamine use.

Adverse event(s) reported in the mother						
Patient ID number	Name of the drug taken by the mother	Dose taken	MedDra term v26	Naranjo score	LCAT	CTCAE v5
1	Duloxetine	30 mg	Somnolence	4	Probable	1
			Libido decreased			
2	Escitalopram	10 mg	Depression aggravated	4	Probable	2
3*	Escitalopram*	5 mg	Raynaud’s phenomenon of the nipple	1	Unlikely	1
4	Escitalopram, Mianserin	10 mg | 10 mg	Anxiety	3	Probable	3
			Insomnia			
5	Opipramole	100 mg	Somnolence	3	Probable	1
6	Sertraline	50 mg	Anxiety	2	Possible	1
7	Sertraline	50 mg	Headache	3	Probable	1
8	Sertraline	100 mg	Lowered blood pressure	5	Probable	1
9	Sertraline, Mianserin, Quetiapine	50 mg | 20 mg | no data	Nusea	4	Probable	1
			Disorientation			
10	Sertraline	50 mg	Nusea	5	Probable	3
			Stomach pain			
11	Sertraline	50 mg	Nusea	4	Probable	2
			Stomach pain			
12	Sertraline	50 mg	Diarrhea	3	Possible	1
13	Escitalopram	10 mg	Lactation decreased	2	Unlikely	1
14	Fluoxetine	20 mg	Lactation decreased	1	Unlikely	1
15	Sertraline	50 mg	Excess lactation	2	Probable	1
16	Sertraline	150 mg	Lactation decreased	2	Unlikely	1
17	Sertraline	50 mg	Excess lactation	3	Probable	1

**TABLE 4:** This table details 9 cases where AEs were reported among infants. Patient ID numbers refers to the mothers. Cases 12,13 and 17 had AEs reported in maternal domain (see Table 3). Each case includes the name of the drug taken by the mother, dose taken by the mother at the moment the AE ocurred, child’s age at that point, breastmilk share in child’s diet, a a description of the adverse event using MedDRA terms (version 27). Each case was assessed for causality using both the Naranjo algorithm (Naranjo et al., 1981) the LCAT tool (Gallagher et al., 2011), and graded using the CTCAE scale.

Adverse event(s) reported in the babies								
Patient ID number	Name of the drug taken by the mother	Dose taken by he mother	Child’s age	Breastmilk share in child’s diet (%)	MedDra term v26	Naranjo score	LCAT	CTCAE v5
12	Sertraline	50 mg	3 weeks	100	Sinus tachycardia	8	Probable	3
13	Escitalopram	10 mg	18 months	25	Change in sleep pattern	2	Unlikely	1
17	Sertraline	50 mg	2 weeks	100	Somnolence	3	Unlikely	2
					Breast feeding problem (infant)			
18	Paroxetine*	20	newborn	100	Somnolence	3	Unlikely	1
19	Sertraline	25 mg	newborn	100	Somnolence	−1	Unlikely	1
					Breast feeding problem (infant)			
20	Sertraline, Hydroxyzine	50 mg | 10 mg	1 month	75	Infant colic	3	Possible	1
					Myoclonic jerks		Unlikely	
21	Sertraline	50 mg	1 month	100	Infant colic	3	Possible	1
22	Sertraline	50 mg	newborn	100	Infant colic	1	Possible	1
22	Sertraline	25	2.5 months	100	Infant colic	2	Unlikely	1

#### 3.6.1 Maternal AEs

Among the 36 reported AEs, 22 (61%) were maternal AEs, encompassing somnolence, decreased libido, worsened depression, Raynaud’s phenomenon of the nipple, anxiety, insomnia, headache, nausea, lowered blood pressure, disorientation, stomach pain, diarrhea, excess lactation, and decreased lactation. Notably, 15 of these maternal AEs were deemed “probable”, have been previously reported by the manufacturer in SmPCs and occurred shortly after drug administration.

In one case the potential causality of Raynaud’s phenomenon of the nipple was evaluated for both medications the mother was taking concurrently: escitalopram and ergotamine. Utilizing the Naranjo and LCAT tool, ergotamine scored higher, suggesting a higher likelihood of causality compared to escitalopram.

The majority of adverse events (AEs) reported were mild and did not necessitate interventions in most patients (n = 17). However, three mothers required specific actions due to AEs. In two instances, a medication dose adjustment was implemented as a consequence of the experienced AEs (in the case of lowered blood pressure and in the case of nausea and stomach pain during sertraline treatment). In the other case, the timing of the doses and the second drug was changed, and then the AEs resolved over time (anxiety and insomnia during escitalopram and mirtaza-pine treatment).

Five mothers (10.6%) reported alterations in lactation during treatment. Two of these mothers additionally reported concurrent adverse events (AEs) in their infants.

A causal relationship between the maternal medication and lactation changes was deemed unlikely in three cases. In the remaining two cases, the medication was considered a probable cause for increased milk production.

#### 3.6.2 Infant AEs

In this study, a total of 12 adverse events (AEs) were identified in nine breastfed infants. Reported infant AEs included change in sleep patterns (n = 1), somnolence (n = 3), breastfeeding difficulties (n = 2), colic (n = 4), myoclonic jerks (n = 1) and single case of a serious adverse event (SAE) - sinus tachycardia requiring hospitalization (n = 1).

This SAE in the infant was categorized as “probable” by both the Naranjo algorithm (score 8) and LCAT tool, indicating a potential but not definitive link to the mother’s medication. However, two consulting physicians at the hospital expressed strong confidence in the causal relationship. A specific genetic predisposition in the infant may have been a contributing factor.

No other AE in infants was categorized as “probable.” Colic was the most common AE, in three instances possibly linked to maternal medication. A neurologist excluded myoclonic jerks as a true AE in one case and other AEs were unlikely caused by maternal medications.

With the exception of one aformentioned case requiring medical attention (sinus tachycardia in an infant), none of the reported adverse events necessitated medical intervention.

## 4 Discussion

Existing data explores DRPs in various settings (Chang et al., 2001; Blix et al., 2004; Bell et al., 2006; Smedberg et al., 2016; Ni et al., 2021) but there is no research specifically focused on breastfeeding women with depressive spectrum disorders (DSDs) in community setting. Our study addresses this gap, contributing valuable data to an under-represented population.

Our findings differ from existing studies: we observed leading DRP: “Effect of drug treatment not optimal” vs. “Treatment safety” in others (Ni et al., 2021) and we did not record any case of unnecessary drug treatment among our patients (possibly due to our younger population and less polytherapy).

Our analysis identified medication non-adherence (the patients did not take the medicaton at all or took lower doses) as the primary factor contributing to ineffective treatment. This highlights the critical importance of prioritizing medication adherence concordance assessment during medical consultations for breastfeeding mothers with DSD. Concordance assessment can help identify potential barriers to adherence and facilitate collaborative decision-making around medication use, ultimately aiming to prevent DRPs at both the prescribing level and the dispensing level.

Evidence-based data endorse the feasibility of considering treatment for DSDs during breastfeeding (Uguz, 2021). Sprague et al. (2020) and Uguz (2021) highlight the availability of several low-risk medications suitable for this population. This study further contributes to that by outlining specific medications employed by its participants, aligning with existing recommendations.

Our study further confirms that while adverse events during DSD treatment during breastfeeding may occur, they are typically mild and require no intervention, as shownd by Hale and Krutsch’s 2023 manual. Considering the current understanding of DSD medication risks and benefits during lactation (Uguz, 2021), the advantages of achieving lactation goals, and the potential risks of early breastfeeding cessation or no breastfeeding at all for both mothers and infants (Perez-Escamilla et al., 2023), the overall risk-benefit analysis strongly favors combined treatment and breastfeeding without additional interventions. This should be carefully considered when developing individualized care plans for lactating mothers receiving DSD treatment.

Lactating women struggling with DSD might be in need of assistance throughout the pharmacotherapy in order to ensure sufficient adherence with respect to patient needs and lactation goals. Maternal uncertainties and anxieties concerning drug safety in lactation need to be taken into account. Our study suggests that pharmacist-led interventions may have the potential to improve DRPs resolution rates in this population.

Interventions for resolving DRPs demonstrably vary across studies due to differences in research setting, identified problem, and patient-specific factors (Bell et al., 2006; Thompson et al., 2015; Smedberg et al., 2016; Abbot et al., 2020; Ni et al., 2021). Abbot et al. (2020) highlights inconsistencies in previous research related to DRPs, attributing them to diverse adherence assessment tools and insufficient reporting details. They emphasized the need for further studies focusing on specific patient populations with standardized, universally comparable tools, categories, and criteria.

Our study addresses this gap by employing a standardized approach within a defined patient population. This includes the use of validated, well-established and recognized tools for DRP categorization (PCNE DRP 9.1) and AEs description and causality assessment, such as MedDRA, Naranjo Scale, LCAT, and CTCAE.

The implemented consultation procedure facilitated the collection of comprehensive data effectively at the early stage of the process incorporating the MILC questionnaire specific to the targeted patient group, and further beyond the initial communication, enabling the assessment of intervention effectiveness and patient outcomes over time.

This study has limitations. The sample size of 47 breastfeeding patients is relatively small, therefore generalizability of our findings may be limited. Incomplete follow-up data from six participants is another limitation. While we were able to collect complete data on their initial interviews, consultations, and other relevant information, we were unable to determine the final consultation outcomes (resolution of DRPs) for these six patients. This limits our ability to fully assess the effectiveness of the pharmacist interventions (a secondary study goal) for the entire population.

Future research directions could involve expanding the study population to encompass larger, more diverse, and multicultural cohorts. This could be coupled with a comparative analysis of DRPs experienced by two distinct populations: breastfeeding mothers with DSD and mothers with DSD who are not breastfeeding. Our study suggests that pharmacist-led interventions in this group of patients may have the potential to improve medication adherence, resolve DRPs, and consequently contribute to enhanced long-term breastfeeding success and maternal mental health. However, these potential benefits require confirmation through further research with a larger, more representative sample and a longer follow-up period to establish robust causal inferences.

## 5 Conclussion

We explored previously uninvestigated factors influencing depressive spectrum disorders treatment in lactating woman. Our findings revealed a high prevalence of drug-related problems among breastfeeding patients, with the most common issue of drug effect not being optimal, manifested by mostly medication non-compliance.

This study sheds light on the unique challenges encountered by lactating patients and healthcare professionals regarding medication adherence and concordance in the therapeutic process. It also provides valuable insights into potential interventions and tools for pharmacists to consider when managing the pharmaceutical care of this specific patient population.

Our findings emphesize the need for a proactive interprofessional approach to DRPs identification and management, particularly for vulnerable populations like breastfeeding mothers. Utilizing available risk-benefit assessment tools during medication selection is crucial for optimizing therapy and ensuring patient safety. Collaboration between pharmacists and other healthcare providers can further enhance medication decision-making in this population.

## Data Availability

The original contributions presented in the study are included in the article/Supplementary Material, further inquiries can be directed to the corresponding author.
