# Supplementary material for: Drug-related problems among breastfeeding patients treated for depressive spectrum disorders
Source: Front Pharmacol. 2024 Jul 23;15:1440681. doi: 10.3389/fphar.2024.1440681 (PMC11300373; doi:10.3389/fphar.2024.1440681)
Supplement: Supplementary file 1 [file Table1.pdf]

Supplementary Material

1 Table 1

List of medications prescribed to registered patients, along with their parameters in relation to lactation, impact on breastfed children, comments and data from SmPC (ATC code - Anatomical Therapeutical Chemical Code, SSRI - Selective serotonin reuptake inhibitor, SNRI - serotonin and norepinephrine reuptake inhibitor, NMRI—Non-selective monoamine reuptake inhibitors, RID - Relative Infant Dose)

**Drug (ATC code)**	**Type**	**Possible Effect on lactation**	**Relative Dose**	**RID** *(Hale, T. W., and Krutsch, K. 2023)*	**Theoretical Dose [mg/kg/d]**	**Plasma levels in infants**	**E-Lactancia risk class**	**Hale's Lactation Risk Class** *(Hale, T. W., and Krutsch, K. 2023)*	**Safety Score Commentary** *(Uguz 2021)*	**Reported adverse events in infants**	**Summary of Product Characteristics for each medication**
Citalopram (N06AB04)	SSRI	Galactorrhea *(Citalopram 2006, in Lactmed)*	3.6 - 18.3% *(APILAM 2022a)*	3.56-5.36%	0.024 - 0.048 *(APILAM 2022a)*	Very low or undetectable (APILAM 2022a)	Very Low risk *(APILAM 2022a)*	L2	Acceptable	Isolated cases of transient sleep disturbances, irritability or colic *(Citalopram 2006, in Lactmed)*	Citalopram passes into breast milk. It is estimated that a breastfed infant will receive approximately 5% of maternal dose (weight adjusted). In infants, no or only minor symptoms were observed. Caution is advised.*(Citalopram 20mg Tablets SmPC)*
Duloxetine (N06AX21)	SNRI	Galactorrhea *(Duloxetine 2006 in Lactmed)*	0.3 - 1% *(APILAM 2022c)*	0.12 - 1.12%	0.004 - 0.01 *(APILAM 2022c)*	Very low or undetectable *(APILAM 2022c)*	Very low risk *(APILAM 2022c)*	L3	Possible with caution	None reported related to the use of the drug *(APILAM 2022c; Duloxetine 2006 in Lactmed)*	Transfer of duloxetine to breastmilk was very poor. Estimated daily infant exposure was estimated as 0.14% of the maternal weight adjusted dose. Duloxetine is not recommended for use during breastfeeding, as the safety of duloxetine in infants is unknown. *(Duloxetine 20 mg SmPC)*
Doxylamine (R06AA09)	Antihistamine	Not known	No data *(APILAM 2022b)*	No data	No data	No data	High risk *(APILAM 2022b*	L3	No data	On a telephone survey, 10% of infants whose mothers were on several types of antihistamine medication have suffered of colicky pain and irritability that disappeared without treatment *(APILAM 2022b; Doxylamine 2006 in LactMed)*	Physicochemical data suggest the transfer of doxylamine into the brastmilk. Because newborns may be more sensitive to the effects of antihistamines the risks cannot be excluded. Doxylamine is contraindicated

											during breastfeeding. (Xonovea SmPC)
Escitalopram (N06AB10)	SSRI	Galactorrhea *(Escitalopram 2006 in LactMed))*	1.7 - 9% *(APILAM 2022d)*	5.2-7.9%	0.004 - 0.01 *(APILAM 2022d)*	Very low or undetectable *(APILAM 2022d)*	Very Low risk *(APILAM 2022d)*	L2	Possible	Isolated cases of irritability, sleepiness *(APILAM 2022d; Escitalopram 2006 in LactMed)*	Escitalopram is expected to be excreted in breast milk. For this reason it is not recommended during breastfeeding. *(Escitalopram 20mg SmPC).*
Fluoxetine (N06AB03)	SSRI	Galactorrhea *(Fluoxetine 2006 in LactMed)*	3 - 20.0% *(APILAM 2022e)*	1.6-14.6%	0.01 - 0.06 *(APILAM 2022e)*	Very low or undetectable *(APILAM 2022e)*	Low risk *(APILAM 2022e)*	L2	Possible	Occasional colic, irritability, insomnia, anorexia and decreased weight gain have been reported *(APILAM 2022e, Fluoxetine 2006 in LactMed);*	Fluoxetine and its metabolite norfluoxetine are known to be excreted in breast milk. There have been adverse events in infants. If treatment with fluoxetine is necessary, you should consider discontinuing breastfeeding.*(Fluoxetine 20mg SmPC)*
Fluvoxamine (N06AB08)	SSRI	Galactorrhea *(Fluvoxetine 2006 in LactMed)*	0.3 - 1.6% *(APILAM 2022f)*	0.3 - 1.4%	0.04 *(APILAM 2022f)*	Very low or undetectable *(APILAM 2022f)*	Very low risk *(APILAM 2022f)*	L2	Possible	Isolated case of gastroenteritis *(APILAM 2022f, Fluvoxetine 2006 in LactMed)*	Fluvoxamine is excreted in small amounts into breast milk, so the drug should not be used in breastfeeding women. *(Fevarin 20mg, SmPC)*
Hydroxyzine (N05BB01)	Antihis-tamine	No effect *(APILAM 2022g)*	No data *(APILAM 2022g)*	No data	No data	No data	Low risk *(APILAM 2022g)*	L2	No data	On a telephone survey, 10% of infants whose mothers were on several types of antihistamine medication have suffered of colicky pain and irritability that disappeared without treatment *(APILAM 2022f)*. Sedation *(Hydroxizine 2006 in LactMed)*	Cetirizine, the main metabolite of hydroxyzine, is excreted in human breast milk.Standard studies have not been performed on its secretion to breastmilk. Seriuos side effects have been observed. This drug is contraindicated during lactation. *(Hydroxizine SmPC)*
Lorazepam (N05BA06)	Benzo-diazepine	No effect *(APILAM 2022)*	1 - 4.3% *(APILAM 2022)*	2.6 - 2.9%	0.0003 - 0.002 *(APILAM 2022)*	No data	Very low risk *(APILAM 2022)*	L3	Possible	One case of sedation *(Lorazepam 2006 in Lactmed)*	Lorazepam passes into human milk, affecting newborns/breastfed children of treated mothers. Lethargy and inability to suck have been observed in infants of mothers taking benzodiazepines. *(Lorazepam 1mg SmPC)*
Mianserin (N06AX03)	Tetracyc-lic antidepre ssant	Not known	0.5 - 1.4% *(APILAM 2022h)*	No data	0.003 - 0.012 *(APILAM 2022h)*	Very low or undetectable *(APILAM 2022h)*	Very low risk *(APILAM 2022h)*	No data	No data	No data	Mianserin is only excreted in breast milk in very small quantities. The benefits of using mianserin should be considered in relation to the possible dangerous effects on the fetus or newborn. *(Mianserin 30mg SmPC)*

Mirtazapine (N06AX11)	Tetracyc-lic antidepre ssant	Galactorrhea *(Mirtazapine 2006 in LactMed)*	0.6 - 4.4% *(APILAM 2022i)*	1.6-6.3%	0.005 - 0.02 *(APILAM 2022i)*	Very low or undetectable *(APILAM 2022i,)*	Very low risk *(APILAM 2022i)*	L3	Possible	None reported related to the use of the drug *(APILAM 2022i; Mirtazapine 2006 in LactMed)*	Animal studies and limited human studies indicate that mirtazapine is secreted into the breast milk only in very small amounts. Deciding whether to continue or stop breastfeeding or continue or discontinue mirtazapine therapy should be undertaken after taking into account the benefits of breastfeeding and the benefits of mirtazapine therapy for the mother. *(Mirtazapine 15mg SmPC)*
Opipramole (N06AA05)	Nonse-lective MRI	Not known	No data	No data	No data	No data	No data	No data	No data	No data	Opipramol should not be used during breastfeeding because its active metabolite passes into milk in small amounts. If the use of opipramol is indicated, breastfeeding should be stopped. *(Pramolan 50mg SmPC)*
Oxazepam	Benzo-diazepine	Not known	0.3 - 1.6% *(APILAM 2022)*	0.28-1%	0.002 - 0.005 *(APILAM 2022)*	Low *(APILAM 2022)*	Very low risk *(APILAM 2022)*	L2	Possible with caution	One case of sedation *(Oxazepam 2006 in Lactmed)*	Oxazepam and its metabolites pass into breast milk, therefore breastfeeding should be stopped while taking this drug. *(Oxazepam 10mg SmPC)*
Paroxetine (N05BA04)	SSRI	Galactorrhea *(Paroxetine 2006 in LactMed))*	2 - 5% *(APILAM 2022j)*	1.2 - 2.8%	0.02 *(APILAM 2022j)*	Very low or undetectable *(APILAM 2022j, (Paroxetine 2006 in LactMed)*	Very low risk *(APILAM 2022j)*	L2	Highly Acceptable	Agitation and difficulty feeding, sleepines and irritability have been reported *(Paroxetine 2006 in LactMed)*	Paroxetine is excreted to breastmilk in small amounts. In published studies, paroxetine concentrations in the serum of breastfed infants were undetectable or very small and no clinical effects were observed in these infants. If symptoms are not expected, breastfeeding may be considered. *(Paroxetine 20mg SmPC)*
Quetiapine (N05AH04)	Anti-psychotic	Galactorrhea *(Quetiapine 2006 in LactMed)*	0.1 - 0.43% *(APILAM 2022k)*	0.02 - 0.1%	0.01 - 0.1 *(APILAM 2022k)*	Very low *(APILAM 2022k, (Quetiapine 2006 in LactMed)*	Very low risk *(APILAM 2022k)*	L2	Possible	Drowsinesswas reported in one case *(Quetiapine 2006 in LactMed)*	Based on limited data on the excretion of quetiapine in human milk, the amount quetiapine, which passes into milk when used at therapeutic doses, appears to be inconsistent. Due to the lack of solid data, a decision must be made on whether to discontinue breastfeeding or therapy. *(Quetiapine 25mg SmPC)*

Sertraline (N06AB06)	SSRI	Galactorrhea *(Sertraline 2006 in Lactmed)*	0.003% *(APILAM 2022l)*	0.04 - 2.2%	0.00002 *(APILAM 2022l)*	Very low or undetectable *(APILAM 2022l; Sertraline 2006 in Lactmed)*	Very low risk *(APILAM 2022l)*	L2	Highly acceptable	Beningn neonatal sleep myoclonus, agitation, irritability, diaharrea, sleepeness were reported, none considered a serious adverse reaction. Causation was not estabished in some *(Sertraline 2006 in Lactmed)*	Published data on sertraline concentrations in breast milk indicate that it passes into the milk small amounts. Very low or undetectable serum drug concentration values were generally observed in breastfed infants with a single except for an infant with sertraline levels approximately 50% of those found in mothers (but without any noticeable impact on the health of this infant). There have been no reports of any adverse reactions in breastfed infants, however, the risk of such effects cannot be excluded. Sertraline is not recommenden during breastfeeding. *(Asertin 50mg SmPC)*

References

APILAM (2002). Association for promotion of and cultural and scientific research into breastfeeding. E-Lactancia.Org. Available at: http://www.ELactancia.org/

APILAM (2021). Lorazepam: level of risk for breastfeeding according to E-Lactancia.org. E-Lactancia.Org. Available at: http://www.E-Lactancia.org/breastfeeding/lorazepam/product/(Accessed February 20, 2024).

APILAM (2021). Oxazepam: level of risk for breastfeeding according to E-Lactancia.org. E-Lactancia.Org. Available at: http://www.E-Lactancia.org/breastfeeding/oxazepam/product/(Accessed February 20, 2024).

APILAM (2022a). Citalopram: level of risk for breastfeeding according to E-Lactancia.org. E-Lactancia.Org. Available at: http://www.E-Lactancia.org/breastfeeding/citalopram/product/(Accessed February 20, 2024).

APILAM (2022b). Doxylamine Succinate: level of risk for breastfeeding according to E-Lactancia.org. E-Lactancia.Org. Available at: http://www.E-Lactancia.org/ breastfeeding/doxylamine-succinate/product/(Accessed February 20, 2024).

APILAM (2022c). Duloxetine: level of risk for breastfeeding according to E-Lactancia.org. E-Lactancia.Org. Available at: http://www.E-Lactancia.org/ breastfeeding/duloxetine/product/(Accessed February 20, 2024).

APILAM (2022d). Escitalopram Oxalate: level of risk for breastfeeding according to E-Lactancia.org. E-Lactancia.Org. Available at: http://www.E-Lactancia.org/breastfeeding/escitalopram-oxalate/product/(Accessed February 20, 2024).

APILAM (2022e). Fluoxetine Hydrochloride: level of risk for breastfeeding according to E-Lactancia.org. E-Lactancia.Org. Available at: http://www.E-Lactancia.org/breastfeeding/fluoxetine-hydrochloride/product/(Accessed February 20, 2024).

APILAM (2022f). Fluvoxamine Maleate: level of risk for breastfeeding according to E-Lactancia.org. E-Lactancia.Org. Available at: http://www.E-Lactancia.org/breastfeeding/fluvoxamine-maleate/product/hydrox (Accessed February 20, 2024).

APILAM (2022g). Hydroxyzine: level of risk for breastfeeding according to E-Lactancia.org. E-Lactancia.Org. Available at: http://www.E-Lactancia.org/breastfeeding/hydroxyzine/product/(Accessed February 20, 2024).

APILAM (2022h). Mianserin Hydrochloride: level of risk for breastfeeding according to E-Lactancia.org. E-Lactancia.Org. Available at: http://www.ELactancia.org/breastfeeding/mianserin-hydrochloride/product/(Accessed February 20, 2024).

APILAM (2022i). Mirtazapine: level of risk for breastfeeding according to E-Lactancia.org. E-Lactancia.Org. Available at: http://www.E-Lactancia.org/breastfeeding/mirtazapine/product/(Accessed February 20, 2024).

APILAM (2022j). Paroxetine: level of risk for breastfeeding according to E-Lactancia.org. E-Lactancia.Org. Available at: http://www.E-Lactancia.org/breastfeeding/paroxetine/product/(Accessed February 20, 2024).

APILAM (2022k). Quetiapine Fumarate: level of risk for breastfeeding according to E-Lactancia.org. E-Lactancia.Org. Available at: http://www.E-Lactancia.org/breastfeeding/quetiapine-fumarate/product/(Accessed February 20, 2024).

APILAM (2022l). Sertraline Hydrochloride: level of risk for breastfeeding according to E-Lactancia.org. E-Lactancia.Org. Available at: http://www.E-Lactancia.org/breastfeeding/sertraline-hydrochloride/product/(Accessed February 20, 2024).

Citalopram (2006). in Drugs and lactation database (LactMed®) (National Institute of Child Health and Human Development). Available at: http://www.ncbi.nlm.nih.gov/books/NBK501185/.

Citalopram 20mg Tablets (2024). Summary of product characteristics (SmPC) -(emc). Available at: https://www.medicines.org.uk/emc/product/5160/smpc#gref (Accessed May 26, 2024).

Doxylamine (2006). in Drugs and lactation database (LactMed®) (National Institute of Child Health and Human Development). Available at: http://www.ncbi.nlm.nih.gov/books/NBK500620/.

Drugs and Lactation Database (2006). Drugs and lactation database (LactMed®).National Institute of Child Health and Human Development.
Duloxetine (2006). in Drugs and lactation database (LactMed®) (National Institute of Child Health and Human Development). Available at: http://www.ncbi.nlm.nih.gov/ books/NBK501470/.

Duloxetine 20 mg gastro-resistant capsules, hard (2024). Summary of product characteristics (SmPC) - (emc). Available at: https://www.medicines.org.uk/emc/product/13590/smpc#gref (Accessed May 26, 2024).

Escitalopram (2006). in Drugs and lactation database (LactMed®) (National Institute of Child Health and Human Development). Available at: http://www.ncbi.nlm.nih.gov/books/NBK501275/.

Escitalopram 20mg tablets (2024). Summary of product characteristics (SmPC) -(emc). Available at: https://www.medicines.org.uk/emc/product/7059/smpc#gref
(Accessed May 26, 2024).

Faverin 50 mg Film-coated Tablets (2024). Summary of product characteristics (SmPC) - (emc). Available at: https://www.medicines.org.uk/emc/product/1169/smpc#gref (Accessed May 26, 2024).

Fluoxetine (2006). in Drugs and lactation database (LactMed®) (National Institute of Child Health and Human Development). Available at: http://www.ncbi.nlm.nih.gov/books/NBK501186/.

Fluoxetine 20mg Capsules (2024). Summary of product characteristics (SmPC) - (emc). Available at: https://www.medicines.org.uk/emc/product/11909/smpc#gref (Accessed May 26, 2024).

Fluvoxamine (2006). in Drugs and lactation database (LactMed®) (National Institute of Child Health and Human Development). Available at: http://www.ncbi.nlm.nih.gov/books/NBK501187/.

Hale, T. W., and Krutsch, K. (2023). Hale's medications and mothers' milk 2023: a manual of lactational pharmacology. Twentieth edition. Springer Publishing.

Hydroxyzine (2006). in Drugs and lactation database (LactMed®) (National Institute of Child Health and Human Development). Available at: http://www.ncbi.nlm.nih.gov/books/NBK500985/.

Hydroxyzine - referral Hydroxyzine - referral | European Medicines Agency. (2024) Accessed May 26, 2024, Available at: https://www.ema.europa.eu/en/medicines/human/referrals/hydroxyzine

Lorazepam (2006). in Drugs and lactation database (LactMed®) (National Institute of Child Health and Human Development). Available at: http://www.ncbi.nlm.nih.gov/books/NBK501231/.

Lorazepam 1mg Tablets (2024). Summary of product characteristics (SmPC) - (emc). Available at: https://www.medicines.org.uk/emc/product/6137/smpc#gref (AccessedMay 26, 2024).

Mianserin (2006). in Drugs and lactation database (LactMed®) (National Institute of Child Health and Human Development). Available at: http://www.ncbi.nlm.nih.gov/books/NBK501764/.

Mianserin 30 mg film-coated tablets (2024). Summary of product characteristics (SmPC) - (emc). Available at: https://www.medicines.org.uk/emc/product/2741/smpc#gref (Accessed May 26, 2024).

Mirtazapine (2006). in Drugs and lactation database (LactMed®) (National Institute of Child Health and Human Development). Available at: http://www.ncbi.nlm.nih.gov/books/NBK501188/.

Mirtazapine 15mg Tablets (2024). Summary of product characteristics (SmPC) - (emc). Available at: https://www.medicines.org.uk/emc/product/531/smpc#gref (Accessed May 26, 2024).

Oxazepam (2006). in Drugs and lactation database (LactMed®) (National Institute of Child Health and Human Development). Available at: http://www.ncbi.nlm.nih.gov/books/NBK501244/.

Oxazepam Tablets 10mg (2024). Summary of product characteristics (SmPC) - (emc). Available at: https://www.medicines.org.uk/emc/product/5338/smpc#gref (Accessed May 26, 2024).

Paroxetine (2006). in Drugs and lactation database (LactMed®) (National Institute of Child Health and Human Development). Available at: http://www.ncbi.nlm.nih.gov/books/NBK501190/.

Paroxetine 20mg Tablets (2024). Summary of product characteristics (SmPC) - (emc). Available at: https://www.medicines.org.uk/emc/product/537/smpc#gref (Accessed May 26, 2024).

Quetiapine (2006). in Drugs and lactation database (LactMed®) (National Institute of Child Health and Human Development). Available at: http://www.ncbi.nlm.nih.gov/books/NBK501087/.

Quetiapine 25 mg film-coated tablets (2024). Summary of product characteristics (SmPC) - (emc). Available at: https://www.medicines.org.uk/emc/product/3079/smpc#gref (Accessed May 26, 2024).

Uguz, F. (2021). A new safety scoring system for the use of psychotropic drugs during lactation. Am. J. Ther. 28 (1), e118–e126. doi:10.1097/MJT.0000000000000909

Xonvea 10 mg/10 mg gastro-resistant tablets (2024). Summary of product characteristics (SmPC) - (emc). Available at: https://www.medicines.org.uk/emc/product/14892/smpc#gref (Accessed May 26, 2024).
